# Supplementary material for: Genome-wide identification and analysis of cystatin family genes in Sorghum (Sorghum bicolor (L.) Moench)
Source: PeerJ. 2021 Jan 21;9:e10617. doi: 10.7717/peerj.10617 (PMC7827979; doi:10.7717/peerj.10617)
Supplement: Supplemental Information 1 [file peerj-09-10617-s001.pdf]

Fig. S1. Amino acid sequence of conserved motifs in SbCys proteins.

Motif 1

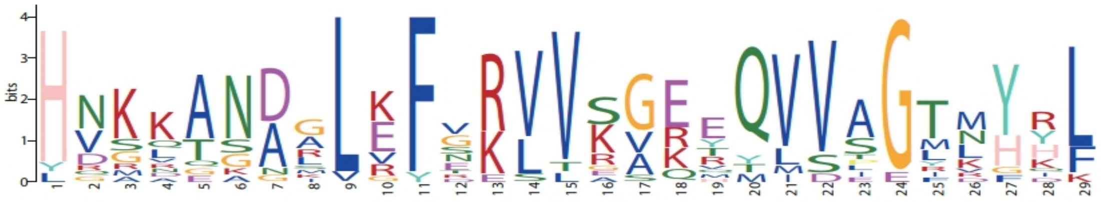

SbCys5	73	2.66e-27	QEVGRWAVSE	HVKKANDGLKFSRVVSGQYQVVEGFNYRL	IIDATDSHGK
SbCys15	87	5.90e-26	IELARFAVDE	HNSKTNAMLEFERLVKVRHQVVAGTMHHF	TVQVKEAGGG
SbCys11	65	5.77e-25	QELARFAVEE	HNKKANALLGYEKLVKAKTQVVAGTMYYL	TVEVKDGEVK
SbCys17	29	1.71e-24	IKLVCFTNAE	HNGKTNAILKFVRSVKVRYQVVAGTMHQF	TVKVKEAGST
SbCys2_2	61	1.71e-24	QELGRWAVSE	HGKQASDRLVFGKVVSGEEQIVAGTKYKL	VIQATRAGAG
SbCys10	63	4.41e-24	DGLGRFAVDE	HNKRENALLEFVRVVEAKEQVVAGTLHHL	TLEAIEAGKK
SbCys16	107	1.51e-23	IELARFAVAE	HNSKTNAALEFVRLVKVRTQLVAGRMYYF	TVEVREVDGG
SbCys2_1	61	8.02e-23	QEIGRWAVSE	HDAAASDSLVFSKVVRGEEQVVAGTNYKL	VIEATKGAGG
SbCys4	141	1.42e-22	QELGGWAVTE	HVRVANDGLRFGEVTSGEQQVVSGMNYRL	VLHATDADGD
SbCys9	84	1.07e-20	QKIARWAVTE	HVMLANDGLKFNKLVSGEVYVSLGYLYHL	IIDASDRDGK
SbCys3	68	1.16e-20	LGGWALGQAK	YQKLAANALRFRRVVRGEEQVVSGMRYRL	YVDAADRAGR
SbCys14	59	3.19e-20	EDAARFAVAH	HNKNQGAALEFTRVLKSKRQVVTGTLHDL	ILEAADAGKK
SbCys13	55	3.34e-19	QYIGRWAVAE	HVKQAKDGLKFDKVVGGEMMMSIGIDFRL	VIDASSTSDG
SbCys12	85	8.74e-17	RFSVAEYNRQ	LRGDGGGRLEFGRVVAAQRQVVSGLKYYL	RVAAVEEGAQ
SbCys1	49	5.56e-16	QQLGRWAVTE	HDKKANDKVKFNRVVSGEETLDPELVGIK	YHLVIDASDG

Motif 2

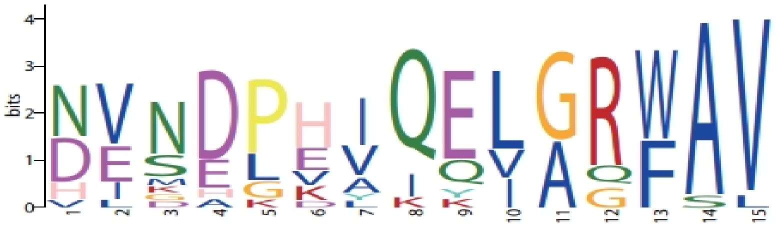

SbCys2_2	44	6.77e-20	PQAVGGWKPI	NVNDPHIQELGRWAV	SEHGKQASDR
SbCys2_1	44	1.99e-18	ENVGRWTPIT	DVNDPEIQEIGRWAV	SEHDAAASDS
SbCys5	56	2.36e-17	LQGGFYQPIV	NINDPHVQEVGRWAV	SEHVKKANDG
SbCys4	124	4.54e-15	PLAGGWSPIR	NVSEPHIQELGGWAV	TEHVRVANDG
SbCys11	48	5.01e-15	LAGGIKDVPA	NENDLHLQELARFAV	EEHNKKANAL
SbCys9	67	1.20e-14	TSTMAETTPI	NVNDPVYQKIARWAV	TEHVMLANDG
SbCys8	97	1.17e-13	PPASNWTPVA	NVKDPKIQQVAQFAV	RIHALSSTEL
SbCys3	49	6.34e-13	PLLGGWNPIP	DVGDKHIQELGGWAL	GQAKYQKLAA
SbCys1	32	8.78e-13	ADSQGWFPLP	DIDAPKVQQLGRWAV	TEHDKKANDK
SbCys13	38	1.80e-12	AQTFGVWYPV	DLSEGHVQYIGRWAV	AEHVKQAKDG
SbCys14	70	1.80e-12	VGDVRDAPEG	HENDLEAIELARFAV	DEHNSKTNAM
SbCys15	64	4.95e-12	KVGARTEVRD	VESDGEVQELGRFSV	AEYNRQLRGD
SbCys16	90	4.18e-11	LCGGVVDAPG	HEMHPDAIELARFAV	AEHNSKTNAA
SbCys7	31	6.85e-11	SAQAAWVPVL	DVNELVIKQVAQFAV	LVYGLAHHRD

Motif3

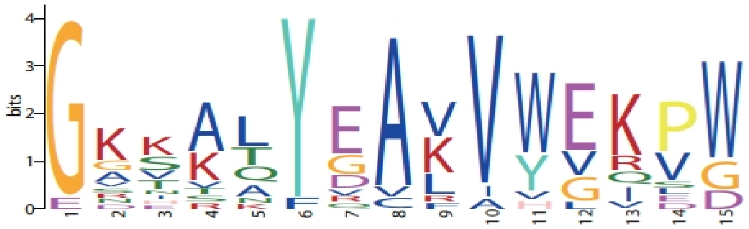

SbCys10	99	2.47e-17	HHLTLEAIEA	GKKKLYEAKVWVKPW	LNFKELQDFS
SbCys15	124	9.24e-16	HFTVQVKEAG	GGKKLYEAKVWEKVW	ENFKQLQSFQ
SbCys14	95	1.05e-14	HDLILEAADA	GKKSLYRAKVWVKPW	EDFKSVVEFR
SbCys5	110	1.94e-14	RLIIDATDSH	GKVAKYEAVVWEKEW	ENFLQLTSFK
SbCys11	101	3.15e-14	YYLTVEVKDG	EVKKLYEAKVWEKPW	ENFKELQEFK
SbCys16	145	7.93e-14	FTVEVREVDG	GASKLYEAKVWLRPW	ENFKGLHAFD
SbCys2_1	99	1.23e-12	LVIEATKGAG	GKIATYGAVVYEKVD	NTRQLLSFAP
SbCys13	93	2.22e-12	LVIDASSTSD	GKHANYEARVHERDW	MDGISLLSFK
SbCys2_2	100	2.69e-12	VIQATRAGAG	GNSATYGAVVYEKVD	KTRQLLSFSP
SbCys8	151	1.40e-11	NYRLLITVSG	GKNTQYEAFVWGILG	TTSWKLLSFT
SbCys7	89	1.53e-11	VVVAARPDDG	GSTAQYDCLVWGVPG	SRSDTWKLRR
SbCys4	178	2.37e-11	RLVLHATDAD	GDVAAYGALVYEQSW	TNTRELMSFA
SbCys3	105	9.88e-11	RLYVDAADRA	GRSVTYVAVIYEQVW	TNTRKLASFT
SbCys12	126	1.01e-9	AAVEEGAQNA	GGERAFDAVVVVKPW	LESRTLLTFA
SbCys6	85	1.46e-9	KMVVTAADAS	GATAQYQVLAWGIPG	TYQWMLLEFK

Motif4

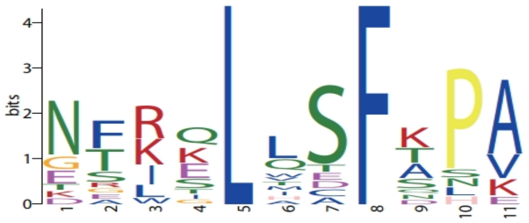

SbCys15	140	8.21e-12	EAKVWEKVWE	NFKQLQSFQPV	GDAAVA
SbCys5	126	1.23e-11	EAVVWEKEWE	NFLQLTSFKPA	N
SbCys2_1	114	9.57e-11	YGAVVYEKVD	NTRQLLSFAPE	N
SbCys2_2	115	1.90e-10	YGAVVYEKVD	KTRQLLSFSPA	N
SbCys11	117	1.34e-9	EAKVWEKPWE	NFKELQEFKPV	EEGASA
SbCys4	194	1.62e-9	GALVYEQSWT	NTRELMSFASA	N
SbCys13	109	1.78e-9	EARVHERDWM	DGISLLSFKPA	K
SbCys8	167	3.71e-9	EAFVWGILGT	TSWKLLSFTPK	Y
SbCys1	104	4.84e-9	YEAVLGEEWI	GRIILISFNPA	R
SbCys3	121	5.28e-9	VAVIYEQVWT	NTRKLASFTLA	SRAH
SbCys9	137	6.27e-9	TAVVRQSTLG	GELSLWSFKPA	N
SbCys12	142	1.57e-8	DAVVVVKPWL	ESRTLLTFAPA	AAK
SbCys10	115	1.85e-8	EAKVWVKPWL	NFKELQDFSHK	GEATTFTNAD
SbCys16	161	5.07e-8	EAKVWLRPWE	NFKGLHAFDPV	ADAA
SbCys17	17	2.30e-7	DTPAGCEKGL	EAIKLVCFTNA	EHNGKTNAIL

Motif5

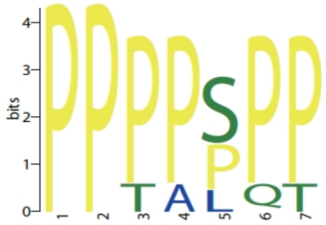

SbCys8	82	1.88e-9	PSPPPPPAYSS	PPPPPPP	ASNWTPVANV
SbCys8	41	1.88e-9	TPSPLPDTPT	PPPPPPP	PESSSTAPPP
SbCys8	69	3.98e-9	SQPPPAASSS	PPPPSPP	PPAYSSPPPP
SbCys15	31	6.94e-9	FPLAVVPVVS	PPPPLPP	GSRRRTASSA
SbCys5	33	2.72e-7	MASRARGGRA	PPTPSPT	QAEIGRLQGG
SbCys8	55	4.61e-7	PPPPESSSTA	PPPASQP	PPAASSSPPP
