# Supplementary figures and images for: Genome-wide identification and analysis of cystatin family genes in Sorghum (Sorghum bicolor (L.) Moench)
Source: PeerJ. 2021 Jan 21;9:e10617. doi: 10.7717/peerj.10617 (PMC7827979; doi:10.7717/peerj.10617)

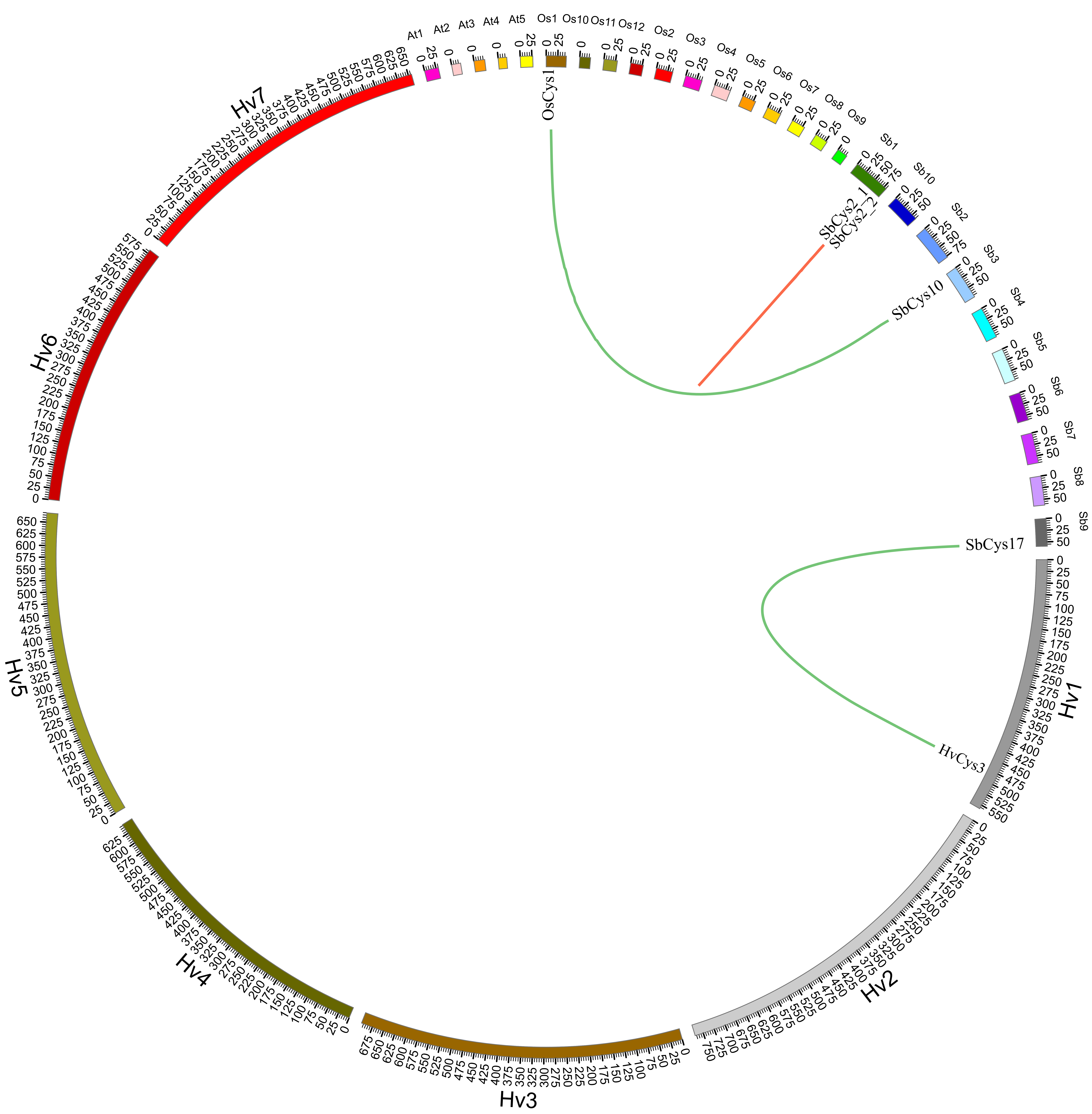

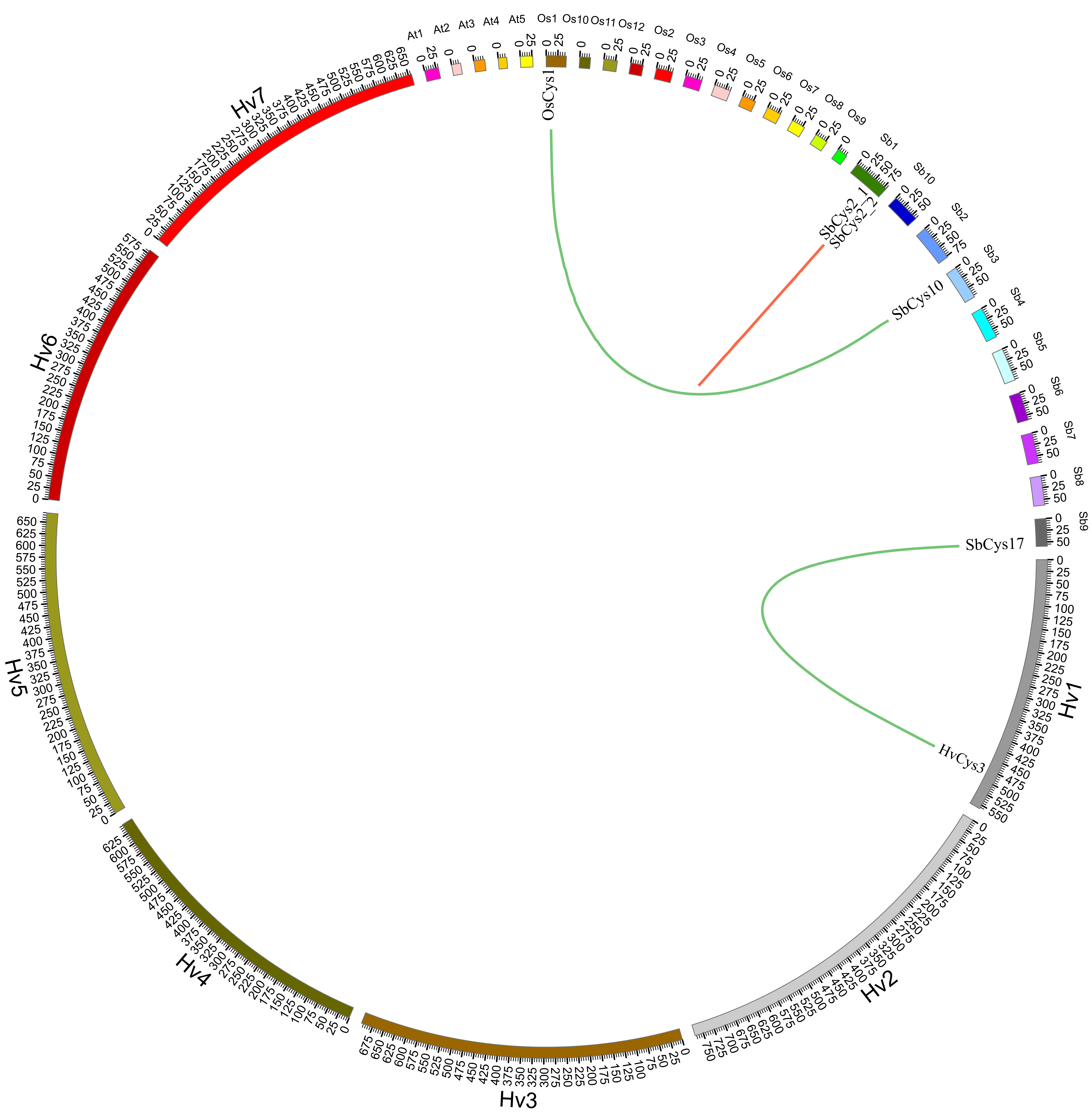

Supplement: Supplemental Information 2 — Green lines connecting two chromosomal regions indicated syntenic regions between rice and sorghum, barley and sorghum. Red lines denoted tandem duplication in Sorghum chromosome. [file peerj-09-10617-s002.pdf]

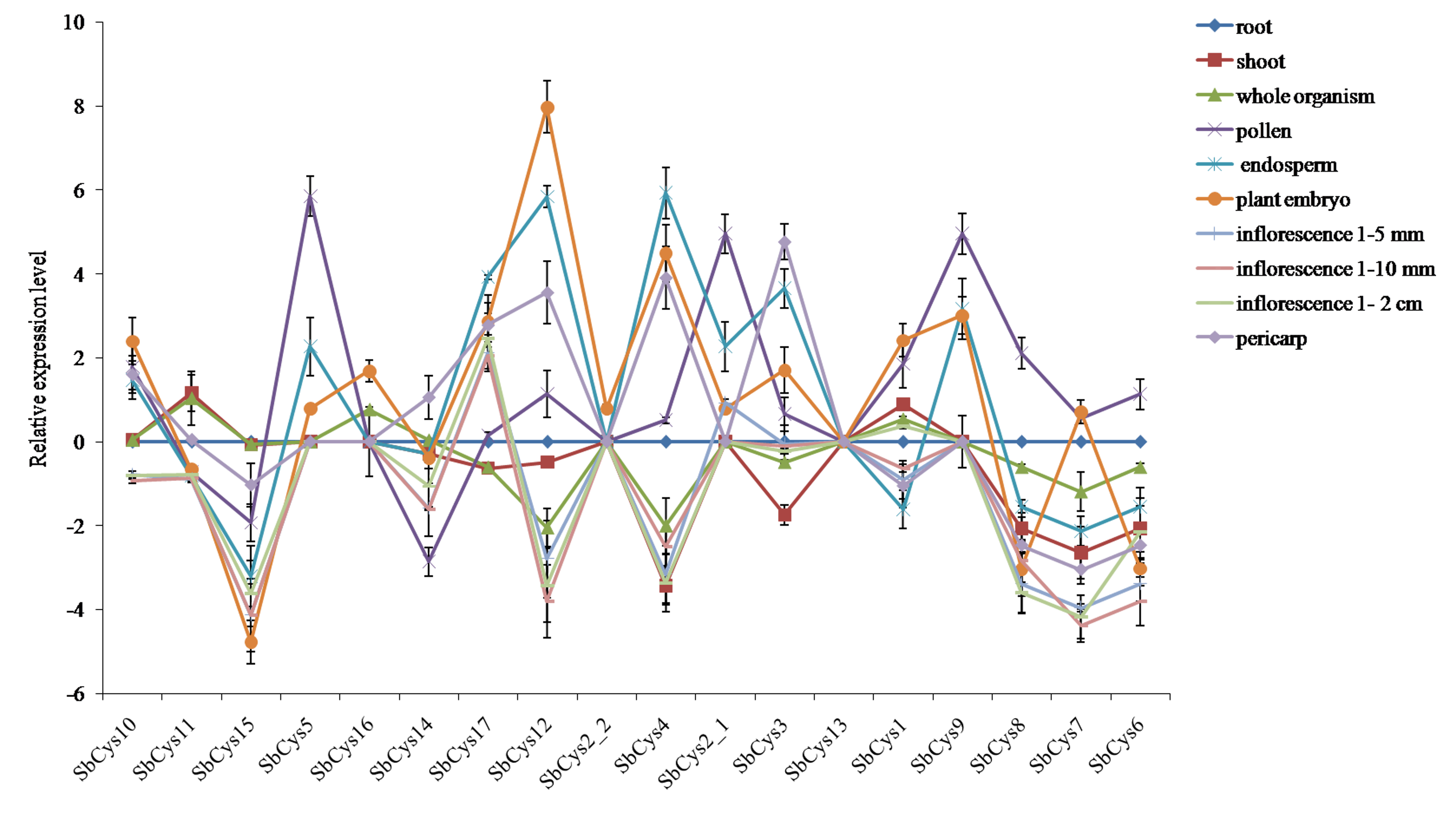

Supplement: Supplemental Information 3 — The data represented fold change (logFC value). [file peerj-09-10617-s003.png]
