# Supplementary material for: Genome-wide identification and analysis of cystatin family genes in Sorghum (Sorghum bicolor (L.) Moench)
Source: PeerJ. 2021 Jan 21;9:e10617. doi: 10.7717/peerj.10617 (PMC7827979; doi:10.7717/peerj.10617)
Supplement: Supplemental Information 5 [file peerj-09-10617-s005.docx]

**Table S2 Primer sequences used for qRT-PCR.**

| Accession number | Forward primer (5’-3’) | Reverse primer (5’-3’) | qRT-PCR efficiency (%) |
| --- | --- | --- | --- |
| SbCys10 | CTCTACGAGGCCAAGGTCTG | CAGCATCTTTGACCACAGGA | 94.5 |
| SbCys11 | AACATCGAATCGTCCCACTC | GCCTTCTTGTTGTGCTCCTC | 92.7 |
| SbCys15 | GAGCACAACAGCAAGACCAA | CACACCTTCTCCCACACCTT | 99.3 |
| SbCys14 | GGGTGCTGCTTTGGAGTTTA | TCCTCTTGTGCAATGTCTGC | 95.1 |
| SbCys17 | AAGCTCGTGTGCTTCACAAA | GGCCTTGTACAGCTTCTTGG | 94.7 |
| SbCys12 | TACAACCGCCAGCTTAGAGG | CGACGCGGAGGTAGTACTTG | 92.4 |
| SbCys4 | ACCCCATGGCTACTACGAGA | TGGAGGACGAGCCTGTAGTT | 91.7 |
| SbCys3 | AGGCCAAGTACCAGAAGCTG | TAGATGACGGCGACGTAGG | 98.8 |
| SbCys1 | CCGGTTGAACTTGACCTTGT | TGAGAACCAGCTTCCTGCTC | 93.6 |
| SbCys7 | TGAACGAGCTGGTGATCAAG | CCACACCAGGCAGTCGTA | 91.7 |
| SbCys6 | GTCCAACAGCGAACGACAC | TTTCTTGAACTCCAGCAGCA | 96.5 |
| SbCys5 | GAACCAGCCACCTTGTTGTT | TGAATTTTAGCCCGTCGTTC | 93.4 |
| SbCys2_2 | TGGAAGCCGATTAATGTGAA | CCGTGTCTTGTCGACCTTCT | 97.2 |
| SbCys2_1 | GACCCCGAAATCCAAGAGAT | CCGCGTATTGTCAACCTTCT | 91.6 |
| SbCys8 | AGCTCGACGGAACTCAACAT | AAGGACAAGAGCTTCCACGA | 93.3 |
| SbCys9 | ACCATGGCAGAGACAACTCC | GCGTCGATGATGAGGTGATA | 94.1 |
| SbCys16 | GAGCACAACAGCAAGACGAA | GCATGGAGCCCCTTAAAGTT | 96.8 |
| SbCys13 | GGCTCAAGTTCGACAAGGTC | CCGGCTTAAATGACAGGAGA | 95.3 |
| AB181991^*^ | GTGTCGCACCAGAGGATCAT | CGCTGGCATACAAGGACAGA | 95.7 |

AB181991^*^: Accession number of reference gene β-actin
